# Cell-free protein synthesis with technical additives – expanding the parameter space of in vitro gene expression

**DOI:** 10.3762/bjoc.20.192

**Published:** 2024-09-04

**Authors:** Tabea Bartsch, Stephan Lütz, Katrin Rosenthal

**Affiliations:** 1 Department of Biochemical and Chemical Engineering, TU Dortmund University, Emil-Figge-Straße 66, 44227 Dortmund, Germany; 2 School of Science, Constructor University, Campus Ring 6, 28759 Bremen, Germany

**Keywords:** cell-free protein synthesis, cGAS, *Escherichia coli* cell-free extract, sfGFP, TX-TL

## Abstract

Biocatalysis has established itself as a successful tool in organic synthesis. A particularly fast technique for screening enzymes is the in vitro expression or cell-free protein synthesis (CFPS). The system is based on the transcription and translation machinery of an extract-donating organism to which substrates such as nucleotides and amino acids, as well as energy molecules, salts, buffer, etc., are added. After successful protein synthesis, further substrates can be added for an enzyme activity assay. Although mimicking of cell-like conditions is an approach for optimization, the physical and chemical properties of CFPS are not well described yet. To date, standard conditions have mainly been used for CFPS, with little systematic testing of whether conditions closer to intracellular conditions in terms of viscosity, macromolecules, inorganic ions, osmolarity, or water content are advantageous. Also, very few non-physiological conditions have been tested to date that would expand the parameter space in which CFPS can be performed. In this study, the properties of an *Escherichia coli* extract-based CFPS system are evaluated, and the parameter space is extended to high viscosities, concentrations of inorganic ion and osmolarity using ten different technical additives including organic solvents, polymers, and salts. It is shown that the synthesis of two model proteins, namely superfolder GFP (sfGFP) and the enzyme truncated human cyclic GMP-AMP synthase fused to sfGFP (t*hs*cGAS-sfGFP), is very robust against most of the tested additives.

## Introduction

In addition to other applications such as biomanufacturing or biosensing, cell-free protein synthesis (CFPS) of enzymes has established itself as a tool for rapid screening of biocatalysts [1–2]. The open environment allows easy manipulation of the protein synthesis [3] and coupling to subsequent enzyme activity assays, e.g., for substrate screening [4–5]. The CFPS system is advantageous for proteins that are difficult to express in a viable host cell, e.g., due to toxic effects on the metabolism [6]. Furthermore, protein synthesis takes only a few hours [7], making the process very fast compared to heterologous expression. CFPS relies on the transcription and translation (TX-TL) system of the donor organism [8]. In addition, the reaction solution contains the DNA-template encoding for the target protein, amino acids and nucleoside triphosphates as substrates, an energy regeneration system and other additives such as polyethylene glycol (PEG) [9].

Although CFPS has been used and improved since the 1960s, there are challenges in its application such as low production volumes, batch-to-batch reproducibility, and reliable kinetic modelling of the system [10–11]. Furthermore, the transferability of CFPS screening results to the cells is limited but important, as in vivo production is often required for preparative scale applications [11–12]. To date, the description of CFPS systems has mainly focused on individual components: the energy regeneration system, the cell extract itself, or individual buffer components [13–14]. For example, the importance of optimizing the concentrations of these reaction components has been demonstrated for four different CFPS systems, in which each system was affected differently by the individual components [15]. This shows that the different CFPS systems have different requirements for their composition, which must be highly balanced. At most, pH and ionic strength are general variables that are considered [13]. Since the main influences of the intracellular environment on the function and cellular behavior of proteins are composition, viscosity, and macromolecular crowding [16], these parameters could have a strong impact on the protein synthesis performance using CFPS. For example, the addition of chemical chaperones, such as alcohols, polyols, polyions or polymers, has a positive effect on protein stability and the soluble fraction expressed with CFPS [17]. The variable composition of CFPS systems with a high number of ingredients and possible reaction conditions [13] thus opens a large parameter space. In addition, non-physiological conditions further extend the parameter space in which CFPS can be performed. This extension would be highly desirable for a coupled CFPS and enzyme assay, where, e.g., an organic solvent is used to solubilize poorly water soluble substrates for the enzyme.

In this study, we therefore aim to fill some of the gaps in the consideration of the general physical properties and potential effects on the performance of *Escherichia coli*-based CFPS. We use technical additives, such as water-soluble macromolecular polymers and salts, which are commonly used as deep eutectic solvents (DES) and extend the properties beyond physiological ranges. We also tested several organic solvents that are miscible and non-miscible with water. For the experiments, two model proteins, the superfolder GFP (sfGFP) and the enzyme human cyclic GMP-AMP synthase-sfGFP, were used, which differ in their size (sfGFP: 27 kDa, t*hs*cGAS-sfGFP: 84 kDa [2,18]) and fractional yield obtained for in vitro expression (sfGFP: 58%; t*hs*cGAS-sfGFP: 9% [12]).

## Results and Discussion

### Effects of additives on fluid properties in CFPS

The fluid properties of the cytoplasm of *E. coli*, the CFPS system, and additives were determined to evaluate their influence on the synthesis performance of CFPS. Polymers, DES, and organic solvents were considered to modify the fluid properties.

#### Polymers and deep eutectic solvents (DES) as additives in CFPS

Polymers (PEG, methylcellulose (MC), and carboxymethylcellulose (CMC)) and DES (choline chloride/urea, betaine/ethylene glycol (EG), and choline chloride/glycerol) were chosen as additives to vary the viscosity, ion concentration, amount of macromolecules, and osmolarity in CFPS. The calculated values for the properties of the CFPS system with polymers and DES added at different concentrations are shown in Table 1 in comparison with the *E. coli* cytoplasm and water.

**Table 1:** Properties of cytoplasm, water, CFPS solution without additives, CFPS with polymers, and CFPS with DES (25 °C, 1 bar). Some values were taken from the literature as indicated. All other values were calculated (Supporting Information File 1). The line marked with the bold text is the standard composition of the in-house CFPS system and serves as reference. PEG, MC, CMC: % 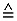 % w/v. Other: % 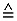 % v/v.

additive to CFPS	concentration [%]	viscosity [mPa·s]	macromolecules [g/L]	inorganic ions [mM]	osmolarity [mOsm]	water content [% v/v]

*E. coli* cytoplasm	n.a.	3–9.7 [16,19]	300–500 [16]	300 [16]	600^a^ [16]	70 [20]
water	n.a.	0.9 [21]	–	–	–	100
PEG-8000	**2** **^b^**	**1.4** [22]	**167–265**	**140**	**405**	**92**
	5	2.2	197–295	140	405	89
	10	8.9 [23]	247–345	140	405	84
methylcellulose	0.5	3.5 [24]	172–270	140	405	92
	0.75	4.7	174–272	140	405	91
	1	6.0 [24]	177–275	140	405	91
	2	12–18^c,d^	187–285	140	405	90
carboxymethylcellulose	0.5	17.8	172–270	140	405	92
	0.75	71.3	174–272	140	405	91
	1	142.5	177–275	140	405	91
	2	1000–1500^c^	187–285	140	405	90
choline chloride/urea 1:2	2	1.5	167–265	232	590	90
	5	1.5	167–265	371	867	87
	10	1.7	167–265	602	1329	82
choline chloride/glycerol 1:2	2	1.4	167–265	214	553	90
	5	1.5	167–265	325	775	87
	10	1.6	167–265	511	1146	82
betaine/ethylene glycol 1:3	2	1.4	167–265	140	405	90
	5	1.5	167–265	140	405	87
	10	1.6	167–265	140	405	82

^a^Sum of inorganic ions and combined metabolites. ^b^Standard composition of in-house CFPS system and reference. ^c^Manufacturer specification. ^d^At 20 °C; n.a.: not applicable.

Comparison of the fluid properties of the natural cytoplasm in *E. coli* [16,19–20] with those calculated for our standard CFPS system shows that although the values are not the same, they are not orders of magnitude different. The CPFS system has a lower viscosity than that of the *E. coli* cytoplasm [25]. The amount of macromolecules in the CFPS system is 167–265 g/L, only slightly less than in a living cell. The calculation is based on the estimated concentrations of macromolecules in the cell extract, tRNA, plasmid, and PEG. Although the molecular weight of PEG-8000 (8000 g/mol) is below the typical definition of a macromolecule (10,000 g/mol [26]), it is conventionally considered an artificial crowding agent [25] and was included in the calculation of macromolecules. The concentration of inorganic ions in the CFPS system, calculated from the magnesium and potassium glutamate concentration, is 140 mM, which is less than half the concentration reported for the cellular environment. The cytoplasmic osmolarity of about 600 mOsm [16] is 50% higher than that calculated for CFPS. Taking into account all defined components, the water content in the CFPS system is 22% higher than in the cytoplasm, as expected for a diluted system.

The fluid properties of the CFPS can be modified by adding various additives. The viscosity of the CFPS system can be increased by adding polymers. Different concentrations of PEG-8000, methylcellulose (MC), and carboxymethylcellulose (CMC) cover a wide range of viscosities including that of the cytoplasm up to a very viscous solution. The concentrations of methylcellulose and carboxymethylcellulose are limited to 2% as the viscosity would have become too high. Simultaneously, polymers contribute to the concentration of macromolecules. PEG, a water-soluble macromolecular polymer, is a commonly used crowding agent to mimic the cellular environment in vitro [25]. Interestingly, it has been observed that different proteins have different PEG-8000 concentration optima [27]. The standard composition of the in-house CFPS system contains 2% PEG-8000, resulting in a lower viscosity of the liquid system. By adding up to 10% PEG-8000, both the viscosity and the concentration of macromolecules in the CFPS system reach the physiological range.

To increase the concentration of inorganic ions, DESs were added to the CFPS solution. Although the viscosities of pure DES are relatively high (choline chloride/urea 1:2: 1200 mPa·s [28], choline chloride/glycerol 1:2: 300 mPa·s [28], betaine/ethylene glycol 1:3: 65 mPa·s [29]), the effect on viscosity when adding 2–10% to the CFPS system is almost negligible. The concentration of inorganic ions in CFPS reactions to which choline chloride was added ranged from 232 to 602 mM, exceeding the cytoplasmic inorganic ion concentration of 300 mM. Osmolarity is also increased by increasing salt concentrations as well. With the addition of 10% choline chloride/urea, the osmolarity of the CFPS system increases to 1329 mOsm. Next to choline chloride/urea and choline chloride/glycerol, betaine/ethylene glycol (EG) was tested as it is considered to be an environmentally friendly natural deep eutectic solvent (NADES) [30] and is widely used with proteins [31]. As it does not consist of any ions there is only a slight increase in the viscosity, but no changes are expected for the other parameters. Except for the water content which, as with all additives, decreases with the percentage of substance added. The lowest value is a water content of 82%, which is still more than 10% above the cytosolic water content of 70% [20], but 10% below the standard conditions of our CFPS system.

#### Solvents as additives in CFPS

For some applications, the usage of solvents in CFPS might be beneficial. Organic solvents as additives do not contribute to more cell-like conditions in CFPS systems, but could allow the use of poorly soluble substrates in combined enzyme assays if they are tolerated.

The effects of organic solvents on the properties of the CFPS system are different from those of polymers and DES. Some fluid properties of the pure solvents and the calculated viscosity of the CFPS system with different concentrations of water-soluble solvents are shown in Table 2. MTBE and *n*-hexane have low solubility in water and formed a second phase on top of the aqueous CFPS solution. For experiments with *n*-hexane and MTBE, the size of the vessel was reduced and the reaction volume was increased to 100 µL to avoid evaporation of the solvent in the headspace of the reaction vessel due to the high vapor pressures. In contrast to the standard volumetric ratio of 20 µL in a 1.5 mL microreaction tube, a visible gradient in the concentration of sfGFP occurred under these conditions. Therefore, shaking at 700 rpm was set to ensure adequate mixing. DMSO and methanol are highly soluble in water, which facilitates handling. Their influence on the viscosity of the CFPS system is negligible, as can be seen in Table 2. The polarities of the different solvents cover a wide range to show the effect on CFPS and give various options for soluble substances.

**Table 2:** Properties of water and added solvents (25 °C, 1 bar). Given values are for pure substances. Viscosities of water-soluble solvents (DMSO and methanol) are additionally calculated for solutions with CFPS at displayed concentrations.

	water	DMSO	methanol	MTBE	*n*-hexane

molecular weight [g/mol]	18.02 [21]	78.14 [32]	32.04 [21]	88.15 [33]	86.18 [21]
density [g/L]	997 [21]	1100^a^ [34]	786 [21]	741 [33]	655 [21]
solubility in water [g/L]	–	1000^b^ [32]	1000 [35]	26^a^ [36]	0.009 [37]
vapor pressure [mmHg]	23.8 [38]	0.6 [32]	127 [35]	245 [33]	153 [37]
viscosity [µPa·s]	890 [21]	2140^a^ [34] 2%: 1409 5%: 1414 10%: 1423	544 [21] 2%: 1411 5%: 1420 10%: 1434	370^c^ [39]	298 [21]
polarity [D]	2.9 [40]	3.96 [41]	2.61 [42]	1.25 [43]	1.08^b^ [44]
log P [–]	–	−1.35^b^ [32]	−0.77^b^ [35]	0.94^b^ [33]	3.9^b^ [37]

^a^At 20 °C. ^b^Temperature unknown. ^c^At 15 °C.

### Effects of technical additives on the CFPS performance

#### In vitro sfGFP production with additives

The in vitro expression of sfGFP, or GFP variants in general, is well established and is often used as a model system for optimization (e.g., with active learning workflows [45]) and performance evaluation. This is convenient because product formation and even concentration can be easily quantified measuring the fluorescence intensity. To take advantage of this, fusion proteins with sfGFP can be constructed for CFPS performance evaluation [46].

**Figure 1:**
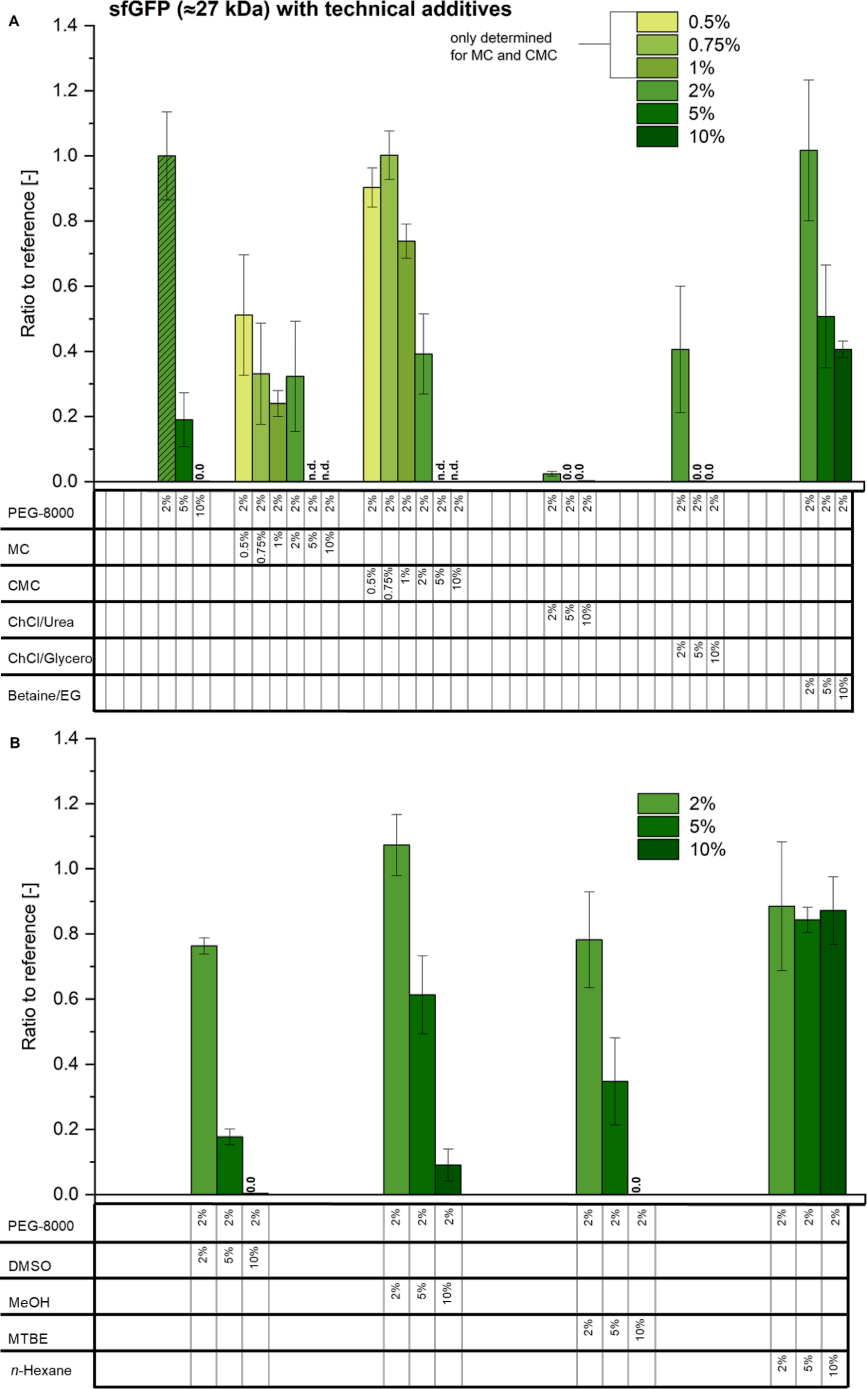
CFPS of sfGFP with different technical additives at various concentrations. Experiments with 2% PEG-8000 serves as reference, equal to 1.77 ± 0.24 mg/mL sfGFP. A) represents the additives polyethylene glycol with a molecular weight of 8000 g/mol (PEG-8000), methylcellulose (MC), carboxymethylcellulose (CMC), choline chloride/urea (ChCl/urea), choline chloride/glycerol (ChCl/glycerol), and betaine/ethylene glycol (betaine/EG); the reference experiment is labeled as shaded bars. B) represents dimethyl sulfoxide (DMSO), methanol (MeOH), methyl *tert*-butyl ether (MTBE), and *n*-hexane. Measurements in triplicates. 0.5–1% only for MC and CMC. PEG, MC, CMC: % 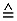 % w/v. Other: % 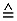 % v/v. Note: 2% PEG-8000 is present as a standard component in all reactions, unless otherwise stated. n.d.: not determined; 0.0: no detectable amount.

Therefore, sfGFP was used to establish a reference CFPS synthesis under standard conditions containing 2% PEG-8000. A concentration of 1.77 mg/mL sfGFP was obtained after 4 hours. The calculated fractional yield of 114% based on the added amino acid concentration is higher than expected, which can be explained either by deviations in the measurements or by the undefined addition of amino acids via the cell-free extract. Irrespective of this, the high concentration of sfGFP obtained shows the high level of optimization of the synthesis. Therefore, it is not expected that the addition of technical additives will further increase the synthesis yield, but the general influence of all additives will be investigated. Figure 1 shows the results of sfGFP synthesis with different technical additives. All values were normalized in relation to the fluorescence intensity of the reference with 2% PEG-8000.

Macromolecular crowding is a known mechanism that positively influences CFPS reactions [25]. Interestingly, increasing the concentration of PEG-8000, and thus increasing the viscosity and macromolecular concentration of the CFPS solution towards the properties of the cytoplasm, has a negative effect on the sfGFP synthesis. This is in agreement with results on the co-optimization of PEG with phosphoenolpyruvate, which indicate an optimal concentration of 4% PEG (7.5 kDa) and a decrease in the activity of the CFPS system at concentrations above 5% [47]. PEG-8000 has been shown to have opposite effects on transcription and translation. While transcription is stable up to concentrations of 10% PEG-8000, translation is already inhibited at a concentration of 1% [48]. When the viscosities are increased with methylcellulose or carboxymethylcellulose, a less negative influence on the sfGFP synthesis is observed. This indicates that the impact of the polymer itself is greater than that of the increased viscosity. A positive effect on the stability and activity of the model enzyme β-ᴅ-glucuronidase by the addition of carboxymethylcellulose has already been reported [49]. However, the effects and states of molecular crowding in the cell are much more complex than what can be mimicked by the sole addition of a polymer. The diffusion of macromolecules depends on the perceived viscosity in the cell, which is inhomogeneous and depends on the location in the cell and its growth phase [50–51].

Comparing the salt concentrations, the concentration in the CFPS system is lower than that in the cells. It was therefore increased by the addition of DES, which have already been used for biological applications [52] and are considered as promising environmentally friendly alternative solvents [53]. The addition of choline chloride appears to have a strong negative effect on the in vitro synthesis of sfGFP. Although the concentration of inorganic ions and osmolarity are close to physiological conditions at a concentration of 2% choline chloride, a reduction of sfGFP production to only 2% was measured for choline chloride/urea and 41% for choline chloride/glycerol compared to the standard composition. This is less than for the addition of any other additive. All other parameters are constant, suggesting that increased salt concentrations have a negative effect on CFPS. Usually, higher salt concentrations can cause an increased precipitation of proteins [54], which would decrease the amount of detectable CFPS product. Other publications describe that the solubility of proteins can be improved with increasing salt concentration by the addition of NaCl [55]. Testing of other salts at high concentrations is necessary to clarify whether the salt concentration or the salt itself is responsible for the low in vitro protein production with choline chloride as an additive.

Interestingly, the addition of organic solvents has little influence on the synthesis performance at concentrations up to 2%. Even methanol concentrations of 5% are well tolerated by the system demonstrating a high robustness against these additives. With *n*-hexane, the amount of sfGFP is stable at around 85% of the reference for all concentrations tested. The reason for this might be that the influence on the CFPS system does not increase with a higher amount of *n*-hexane due to the low solubility of *n*-hexane in water, a limited interfacial area, and evaporation in the headspace. There is no clear trend for the influence of the polarity or log P of the added solvents. Among the water-miscible additives, methanol, whose dipole moment is relatively close to that of water, is better accepted than DMSO.

#### In vitro t*hs*cGAS-sfGFP production with additives

The CPFS system used, or CFPS in general, is not further optimized for the production of t*hs*cGAS-sfGFP or other specific enzymes. By default, GFP is generally used as a model protein to optimize the composition of CFPS, as it provides a simple and robust readout, the fluorescence signal. As a result, the composition of the CFPS solution is optimized for the synthesis of GFP. In addition, larger and more complex enzymes are usually more difficult to synthesize with CFPS [56], although there are exceptions, such as the production of non-ribosomal peptide synthetases with a molecular weight higher than 100 kDa [57]. However, these enzymes are often of great interest for specific applications in biomanufacturing. Cyclic GMP-AMP synthase (cGAS) is one of these enzymes. cGAS and its biocatalytic product 2’3’-cyclic GMP-AMP (cGAMP) are part of the innate immune response in higher eukaryotes [58]. cGAMP is therefore a promising candidate for pharmaceutical applications [59]. The successful synthesis of the fusion protein of truncated human cGAS and superfolder GFP (t*hs*cGAS-sfGFP) using the in-house *E. coli*-based CFPS system has already been demonstrated under standard conditions [18]. We have now repeated this experiment and tested the synthesis of t*hs*cGAS-sfGFP with the addition of additives.

**Figure 2:**
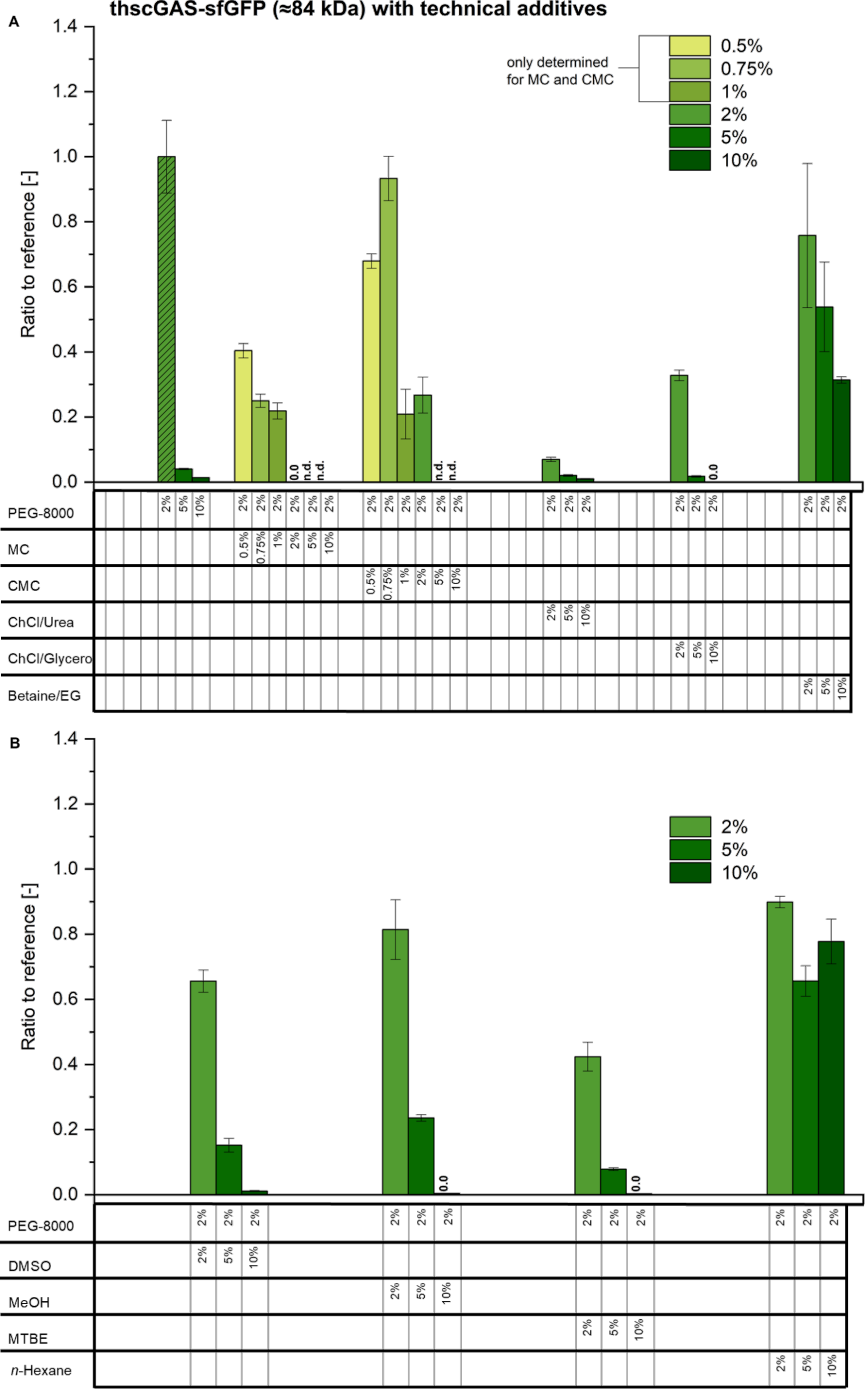
CFPS of t*hs*cGAS-sfGFP with different technical additives at various concentrations. Experiments with 2% PEG-8000 serve as reference, equal to 0.13 ± 0.02 mg/mL t*hs*cGAS-sfGFP. A) represents the additives polyethylene glycol with a molecular weight of 8000 g/mol (PEG-8000), methylcellulose (MC), carboxymethylcellulose (CMC), choline chloride/urea (ChCl/urea), choline chloride/glycerol (ChCl/glycerol), and betaine/ethylene glycol (betaine/EG). The reference experiment is labeled as shaded bars. B) represents dimethyl sulfoxide (DMSO), methanol (MeOH), methyl *tert*-butyl ether (MTBE), and *n*-hexane. Measurements in triplicates. 0.5–1% only for MC and CMC. PEG, MC, CMC: % 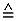 % w/v. Other: % 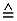 % v/v. Note: 2% PEG-8000 is present as a standard component in all reactions, unless otherwise stated. n.d.: not determined; 0.0: no detectable amount.

The average production of t*hs*cGAS-sfGFP under reference conditions using the in-house CFPS system was 0.13 mg/mL, which is comparable to published data [12]. The fractional yield for t*hs*cGAS-sfGFP is approximately 10.5% and therefore has potential for optimization. Figure 2 summarizes results for the production of t*hs*cGAS-sfGFP. In general, the trend of the protein concentrations obtained is consistent with that observed for sfGFP. The production levels are at or below the reference value of 2% PEG-8000. Among the additives, 0.75% of carboxymethylcellulose and 2% of betaine/EG, methanol and *n*-hexane are the best with 76–93% compared to the reference. The addition of carboxymethylcellulose results in 75% for sfGFP and t*hs*cGAS-sfGFP.

#### Parameter scope and robustness of CFPS

The composition of reaction media for enzymatic applications, especially in the chemical industry, has been expanded to technical additives [60]. It was therefore tested whether CFPS is also compatible with the addition of technical additives. CFPS show a high robustness against various additives including cytotoxic solvents, which was demonstrated by the synthesis of sfGFP and t*hs*cGAS-sfGFP. The combination of CFPS with activity assays would allow further parallelization and also miniaturization, for example into femtoliter-sized droplets [61–62].

In general, protein synthesis with CFPS is best at low additive concentrations. The general trend for the production of t*hs*cGAS-sfGFP were similar compared to sfGFP, but the protein concentrations obtained were overall lower. The protein production still works for most additives up to a concentration of 10%, except when 10% of MTBE or choline chloride/glycerol was added, which completely inhibited the CFPS system. The effect of the addition of *n*-hexane in the experiments is low, which could be due to its low solubility. For the other additives tested, 5–10% seems to be the limit, with the exception of betaine/EG, which is accepted in higher concentrations.

The robustness of the transcription-translation machinery is astonishing and extends the parameter range for CFPS. Successful protein synthesis was observed at very high viscosities, increased concentrations of macromolecules, organic ions, and osmolarity. The tolerated viscosities (determined for standard conditions: 25 °C, 1 bar) range from 1.4 to about 1000 mPa·s. At concentrations of inorganic ions of up to 602 mM and an osmolarity of 1329 mOsm, small concentrations of t*hs*cGAS-sfGFP were still detected. The concentration of macromolecules and the water content of the standard CFPS system are in the range of *E. coli* cytoplasm [16,20]. We were able to extend these conditions for the CFPS system to values between 167 and 265 g/L macromolecules corresponding to 82 to 92% of water content.

## Conclusion

This study shows that CFPS is robust against various technical additives. The general trend is a decrease in protein concentration with increasing concentrations of additives, but still detectable amounts of product were reported with 10% of PEG, choline chloride/urea, betaine/ethylene glycol, DMSO, methanol, and *n*-hexane. The results are most promising for betaine/ethylene, methanol, and *n*-hexane and open new potential for applications such as on-site synthesis of enzymes for subsequent biotransformation. In addition to evaluating the physical properties of a standard CFPS system, the range of parameters for CFPS was successfully extended to high values of viscosity, concentrations of inorganic ions, and osmolarity. Carboxymethylcellulose was identified as an interesting alternative crowding agent. This provides a starting point for a multifactorial approach to optimize the synthesis of non-model enzymes.

## Experimental

### Additives and preparation of deep eutectic solvents

The selected additives are supposed to shift the properties of the reaction solution in more extreme directions or are interesting for other reasons. PEG-8000 (Sigma, Darmstadt, Germany) is a molecular crowder that is used in the CFPS system by default. Carboxymethylcellulose sodium salt (Roth, Karlsruhe, Germany) and methylcellulose (VWR, Darmstadt, Germany) are polymers that expand the viscosity range. Choline chloride (VWR, Darmstadt, Germany) + urea (Roth, Karlsruhe, Germany) (molar ratio 1:2) and choline chloride + glycerol (Roth, Karlsruhe, Germany) (molar ratio 1:2) were chosen to increase the amount of salts in the solution. Betaine (Sigma, Darmstadt, Germany) + ethylene glycol (Roth, Karlsruhe, Germany) (molar ratio 1:3) was selected as additional common DES. The DES were prepared by weighting out the substances and stirring at up to 100 °C until liquid state was reached [29]. For the organic solvents, DMSO (Roth, Karlsruhe, Germany) and methanol ≥99% (Roth, Karlsruhe, Germany) were used as water-soluble, and MTBE (Arcos organics, Schwerte, Germany) and *n*-hexane (Lach:ner, Neratovice, Czech Republic) as non-water-soluble solvents. Values for properties were taken from the databases Chemistry WebBook by the National Institute of Standards and Technology (NIST) (https://webbook.nist.gov/) at 1 bar and 25 °C, PubChem by the National Center for Biotechnology and Information (https://pubchem.ncbi.nlm.nih.gov/), and GESTIS-Stoffdatenbank by Institut für Arbeitsschutz der Deutschen Gesetzlichen Unfallversicherung (https://gestis.dguv.de/), datasheets provided by manufacturers and other sources as referenced at the corresponding point. Viscosity of the standard CFPS system was estimated as that of 2% PEG in water. For the other concentrations and polymers, the value was assumed as that for the component with pure water as well, as the contribution of the other components to the viscosity is considered neglectable in comparison to the high viscosity of the polymer–water mixtures. Some of these were inter- or extrapolated from published values. For carboxmethylcellulose values were derived from the manufacturer’s specification with the rule of thumb that doubling the concentration increases the viscosity by a factor of about 8 [63]. As a simple approach according to Arrhenius, the viscosity for mixtures with DES and the water-soluble solvents was calculated with Equation 1 [64], as only minor influences are assumed at the concentrations used in this work:


[1] logηs=N1logη+1N2logη2

The concentration of macromolecules was calculated based on the cytosolic composition and the average OD_600_ at the harvest of the culture for the cell-free extract. With the derived number of cells, the cellular volume of 4.4 µm³ per *E. coli* cell [65] and the intracellular concentration of macromolecules, the range for the total amount of macromolecules extracted from the culture was determined. The volume of buffer, dilution at CFPS assembly and PEG-8000, tRNA and plasmid as further macromolecules were included for the calculation of the macromolecular concentration of the CFPS mix.

Magnesium and potassium glutamate were considered as the contributing inorganic ions for the reference composition, for the DES corresponding salts were added to the value of 140 mM.

For the osmolarity the concentrations of all defined components were multiplied with their number of dissociated particles, which was assumed as 1 for most components and 2 for magnesium and potassium glutamate and combined with the calculated concentration of macromolecules.

For the water content, the amount of all known components was subtracted from the 100% of pure water, additives decreased that value by the percentage of their contribution. Calculations and further details can be found in Supporting Information File 1.

### Cell-free protein synthesis

The CFPS system was prepared and the reactions performed according to Rolf et al. [18] with the described strains *E. coli* BL21(DE3) pAR1219 for extract preparation and *E. coli* DH5α pETH6sfGFP and *E. coli* DH5α pETSUMOt*hs*cGASGFP for plasmid production. Minor variations are stated in the following. The preculture for extract preparation was grown for 20 h at 200 rpm and 37 °C, centrifugation for cell harvesting and washing was performed for 20 min at 3220*g* and storage was at −70 °C. The extract contained 46–67 mg/mL protein. It was premixed with the buffer consisting of magnesium and potassium glutamate, 20 amino acids, HEPES, ATP, GTP, cytidine triphosphate (CTP), uridine triphosphate (UTP), tRNA, coenzyme A (CoA), nicotinamide adenine dinucleotide (NAD), cyclic adenosine monophosphate (cAMP), folinic acid, spermidine, 3-PGA, and PEG-8000 to obtain a master mix. The master mix was assembled with the plasmid encoding for sfGFP respectively t*hs*cGAS-sfGFP and nuclease-free water, which added up the free volume to the final CFPS volume of 20 µL in a 1.5 mL microreaction tube. The final composition of the reaction was 11–16 mg/mL protein from extract, 10 mM magnesium glutamate, 130 mM potassium glutamate, 1.5 mM of each of 20 amino acids except for leucine, which is 1.25 mM, 50 mM HEPES, 1.5 mM ATP and GTP, 0.9 mM CTP and UTP, 0.2 mg/mL tRNA, 0.26 mM CoA, 0.33 mM NAD, 0.75 mM cAMP, 0.068 mM folinic acid, 1 mM spermidine, 30 mM 3-PGA, 2% PEG-8000, and 1 nM plasmid DNA. Reactions were incubated for 4 h at 37 °C with no shaking. Resulting fluorescence intensities were measured from 2 µL reaction solution in 98 µL 0.5 M HEPES buffer (pH 8.0) in 384-well microplates with a FLUOstar^®^ Omega multi-mode microplate reader (BMG LABTECH, Ortenberg, Germany). The endpoint measurement was set to a gain of 1390, λ_ex_ 485 nm and λ_em_ 520 nm.

For the experiments with organic solvents, the corresponding volume of 2, 5, and 10% v/v was added right before the incubation. To keep the final volume at 20 µL the volume of added water was decreased by the same volume. For the non-water-soluble solvents, the scale was linearly increased to 100 µL in a 200 µL microreaction tube, incubation was with shaking at 700 rpm. DES and PEG were pre-diluted with nuclease-free water for better pipettablity and added to a final concentration of 2, 5 and 10% v/v, respectively 2, 5 and 10 % w/v for PEG. Methylcellulose and carboxymethylcellulose were added as solid powders to the master mix in the appropriate amount to set the final concentration in the reaction to 0.5, 0.75, 1 and 2% w/v.

All reactions were prepared in triplicates with an additional negative control without the addition of DNA. For all reactions with additives a triplicate of the standard composition was run at the same time and with the same cell-free extract as a reference.

### Correlation of fluorescence intensities and protein concentrations

Plasmids for CFPS were expressed in *E. coli* BL21 (DE3) and purified as described by Rolf et al. [18]. The quantifications of purified proteins and set dilution series were performed with the Bradford assay [66]. Purity of the in vivo produced proteins was checked with sodium dodecyl sulfate polyacrylamide gel-electrophoresis (SDS-PAGE) [67]. Impurities were quantified with ImageJ [68] and measured protein concentrations corrected by the results to gain concentrations of pure sfGFP and t*hs*cGAS-sfGFP. Fluorescence was measured with FLUOstar^®^ Omega multi-mode microplate reader (BMG LABTECH, Ortenberg, Germany) under the same conditions as for the in vitro*-*produced proteins to determine the correlation between fluorescence intensity and protein concentration for each protein.

### Fractional yield

The fractional yield is the ratio between the theoretically achievable protein concentration based on the amount of provided amino acids in a CFPS system and the sequence of the target protein and the experimentally achieved result [12]. Fractional yields in this work have been calculated using the excel sheet provided by Rolf et al. [12] and can be found in Supporting Information File 2 and Supporting Information File 3.

## Supporting Information

File 1 Calculations of viscosity, macromolecules, inorganic ions, osmolarity and water content for CFPS in Table 1.

File 2 Calculations of viscosity for CFPS with water-soluble solvents in Table 2.

File 3 Fractional Yield of average sfGFP production with CFPS.

File 4 Fractional yield of average t*hs*cGAS-sfGFP production with CFPS.

## Data Availability

All data that supports the findings of this study is available in the published article and/or the supporting information to this article.
